# Formation of Boron-Carbon Nanosheets and Bilayers in Boron-Doped Diamond: Origin of Metallicity and Superconductivity

**DOI:** 10.1186/s11671-015-1215-6

**Published:** 2016-01-12

**Authors:** S. N. Polyakov, V. N. Denisov, B. N. Mavrin, A. N. Kirichenko, M. S Kuznetsov, S. Yu Martyushov, S. A. Terentiev, V. D. Blank

**Affiliations:** Technological Institute for Superhard and Novel Carbon Materials, Troitsk Moscow, 142190 Russia; Institute of Spectroscopy, Russian Academy of Sciences, Troitsk Moscow, 142190 Russia

**Keywords:** Boron-doped diamond, B-C nanosheets and bilayers, 2D misfit layer structure, Electronic band structure, Mott and superconducting transitions, 62.23.St, 74.25.nd, 63.22.Np, 61.05.cp

## Abstract

The insufficient data on a structure of the boron-doped diamond (BDD) has frustrated efforts to fully understand the fascinating electronic properties of this material and how they evolve with doping. We have employed X-ray diffraction and Raman scattering for detailed study of the large-sized BDD single crystals. We demonstrate a formation of boron-carbon (B-C) nanosheets and bilayers in BDD with increasing boron concentration. An incorporation of two boron atoms in the diamond unit cell plays a key role for the B-C nanosheets and bilayer formation. Evidence for these B-C bilayers which are parallel to {111} planes is provided by the observation of high-order, super-lattice reflections in X-ray diffraction and Laue patterns. B-C nanosheets and bilayers minimize the strain energy and affect the electronic structure of BDD. A new shallow acceptor level associated with B-C nanosheets at ~37 meV and the spin-orbit splitting of the valence band of ~6 meV are observed in electronic Raman scattering. We identified that the superconducting transitions occur in the (111) BDD surfaces only. We believe that the origin of Mott and superconducting transitions is associated with the two-dimensional (2D) misfit layer structure of BDD. A model for the BDD crystal structure, based on X-ray and Raman data, is proposed and confirmed by density functional theoretical calculation.

**Electronic supplementary material:**

The online version of this article (doi:10.1186/s11671-015-1215-6) contains supplementary material, which is available to authorized users.

## Background

A boron-doped diamond is of great interest because it exhibits a variety of intriguing physical properties, such as semiconductor metal (Mott) [1–3] and superconducting transitions [4]. Although the boron-doped diamond (BDD) has been the subject of many important studies [1–11], full understanding of its structure and physical properties has not yet been realized and the origin of these transitions is the subject of ongoing debate. Obtaining detailed structural information has been hindered by the fact that all superconducting BDD samples were thin chemical-vapor-deposited films, granular [11], and polycrystals [4]. To eliminate these problems, we have grown large-sized, high-quality BDD single crystals and used them to obtain structure and structure-property understanding. There have been doubts about the actual involvement of boron atoms in a true diamond lattice. In fact, an amorphous boron-carbide-type phase between true diamond polycrystals has been suggested as responsible for superconductivity [10].

Some previously published experimental results suggest a two-phase behavior in BDD [12–16]. The lattice constant is practically permanent in the range of boron concentrations from 10^17^ to 10^20^ cm^−3^ according to the X-ray data [12], while that should increase with the increase of the boron concentration due to Vegard law. The question arises about the reason of the lattice constant invariability. Two slopes in the dark conductivity plot of BDD [13] demand an explanation too. Raman bands at 480 and 1230 cm^−1^ were observed by many researchers, but the origin of these bands was still unclear. A two-phase behavior has been observed by Raman spectroscopy at different excitation wavelengths [14]. The observed large, narrow resistance peak in BDD is quite similar to that known in low-dimensional (D) systems (1D or 2D), though the authors interpret it as an unusual behavior in a 3D system [15]. Chemical-vapor-deposited BDD films lose their superconductivity after being polished [16]. The possible reason is 2D surface superconductivity. The idea of a quasi 2D superconductor, which comprises a “sandwich” of a metallic film between dielectric layers, was suggested by Ginzburg [17].

In the present work, we demonstrate that the BDD has a complex structure. It is a conventional 3D crystal with point defects at the low doping level. Raman spectra show the point-defect-induced bands. The X-ray diffraction analysis is not sensitive to point defects. B-C nanosheets start to form at the boron concentration ≥4 × 10^18^ cm^−3^. These nanosheets have a small size and cannot be detected by X-ray diffraction. Raman bands at 480 and 1230 cm^−1^ associated with the B-C nanosheets appear. Simultaneously with the appearance of these Raman bands, the 1*s* → n*s* electronic transitions between the 1*s* ground and n*s* excited acceptor states are observed in electronic Raman spectra of BDD that are also associated with the B-C nanosheets. The analysis of these Raman spectra indicates unambiguously on the appearance of the new shallow acceptor level at ~37 meV and the spin-orbit splitting of the valence band of ~6 meV. The B-C nanosheets are self-assembled into the B-C bilayers at the boron concentration ~2 × 10^20^ cm^−3^. The dimensions and number of B-C bilayers are sufficient to observe satellite peaks and high-order reflections in the X-ray diffraction. The Mott transition occurs in the bulk BDD when the acceptor states merge with the top of the valence band and the 1*s* → n*s* electronic transitions are not already observed in electronic Raman spectra of BDD while the superconducting transitions occur in the BDD surfaces only.

## Methods

### Materials

Type IIb diamond single crystals were grown by the temperature gradient method under high pressure at 5.5 GPa and high temperature at 1440 °C in a «toroid»-type high-pressure apparatus. *Fe–Al–C* alloy (91:5:4 by wt.%) was used as the solvent metal. *Al* was added to the solvent as the nitrogen getter. High-purity (99.9995 %) graphite was used as the carbon source. Amorphous boron powder was added to the carbon source in the wide range of concentrations as the doping agent. The synthetic diamond crystals with sizes of ~0.5 mm and (100) surface orientation were used as a seed. Temperature in the reaction cell during growth run was directly measured by Pt6%Rh–Pt30%Rh thermocouple with an accuracy of ±2 °C. An axial temperature gradient between the carbon source and seed crystal was ~30 °C. Large-sized BDD single crystals were grown in the environment with boron concentrations from 0.01 to 3.61 at.%. BDD plates with (001) and (111) surface orientations were laser-cut from as-grown single crystals and polished.

### Characterization Techniques

BDD plates were characterized by spark source mass spectrometry, X-ray topography, X-ray diffraction, and Raman scattering. X-ray diffraction studies were carried on a Rigaku D/max-RC and fully loaded PANalytical Empyrean diffractometers. A Rigaku X-ray diffraction topography system XRT-100 CCM equipped with an UltraX18 X-ray generator was used for the white beam and traverse topography. The X-ray patterns were recorded by an Imagine Plate, a Medipix2 PIXel^3D^ detector, and a Rigaku X-ray CCD camera. High-resolution X-ray measurements for detection of satellite peaks were carried out with fully open aperture of the PIXel^3D^ detector using a Ge 4×(220) hybrid monochromator as a beam conditioner. The electronic Raman spectra were recorded on a homemade multichannel triple-stage spectrometer equipped with a Spec 10:400B CCD camera with a spectral resolution of 0.6 cm^−1^. A 514-nm line of an Ar^+^–Kr^+^ laser (Spectra-Physics Model 2018-RM) was used to excite the Raman spectra. The Raman spectra were studied at ~20 K using a He cryostat. The vibrational Raman spectra were excited with 514- and 257-nm laser radiations (Spectra-Physics Model 2065-7S with Z-Lok and Wave Train CW Frequency Doubler) and recorded using a Raman spectrometer on the basis of TRIAX-552 spectrograph with a TE-cooled CCD camera SPEX10: 2kBUV and confocal Raman microscope with a spatial resolution of ~1 μm and probing depth of ~2 μm. Boron concentrations were determined by the IMS-01-BM2 spark mass spectrometer. The etalon with the known content of boron atoms implanted in the IIa diamond plate was used for calibration.

### Ab initio DFT Calculations

Ab initio density functional theoretical (DFT) calculations were carried out using Quantum Espresso package (http://www.quantum-espresso.org). Calculations of the phonon density of states (PDOS) and the Eliashberg function for a 10-atom hexagonal cell have been performed in the framework of DFT in the local-density approximation of the exchange-correlation potential. The plane-wave pseudopotential method, implemented in the Quantum Espresso code, was used. Total energy of the system was converged with the accuracy of 10^−10^ Ry for 100 Ry energy cutoff of the plane-wave basis. A sampling of the Brillouin zone was done with the 8 × 8 × 8 Monkhorst-Pack k-point grid for the phonon calculations and 24 × 24 × 24 for the electron-phonon analysis. After the cell optimization, the forces did not exceed 10^−4^ eV/Å and stress in the cell was ~10^−4^ GPa.

## Results and Discussion

**Fig. 1:**
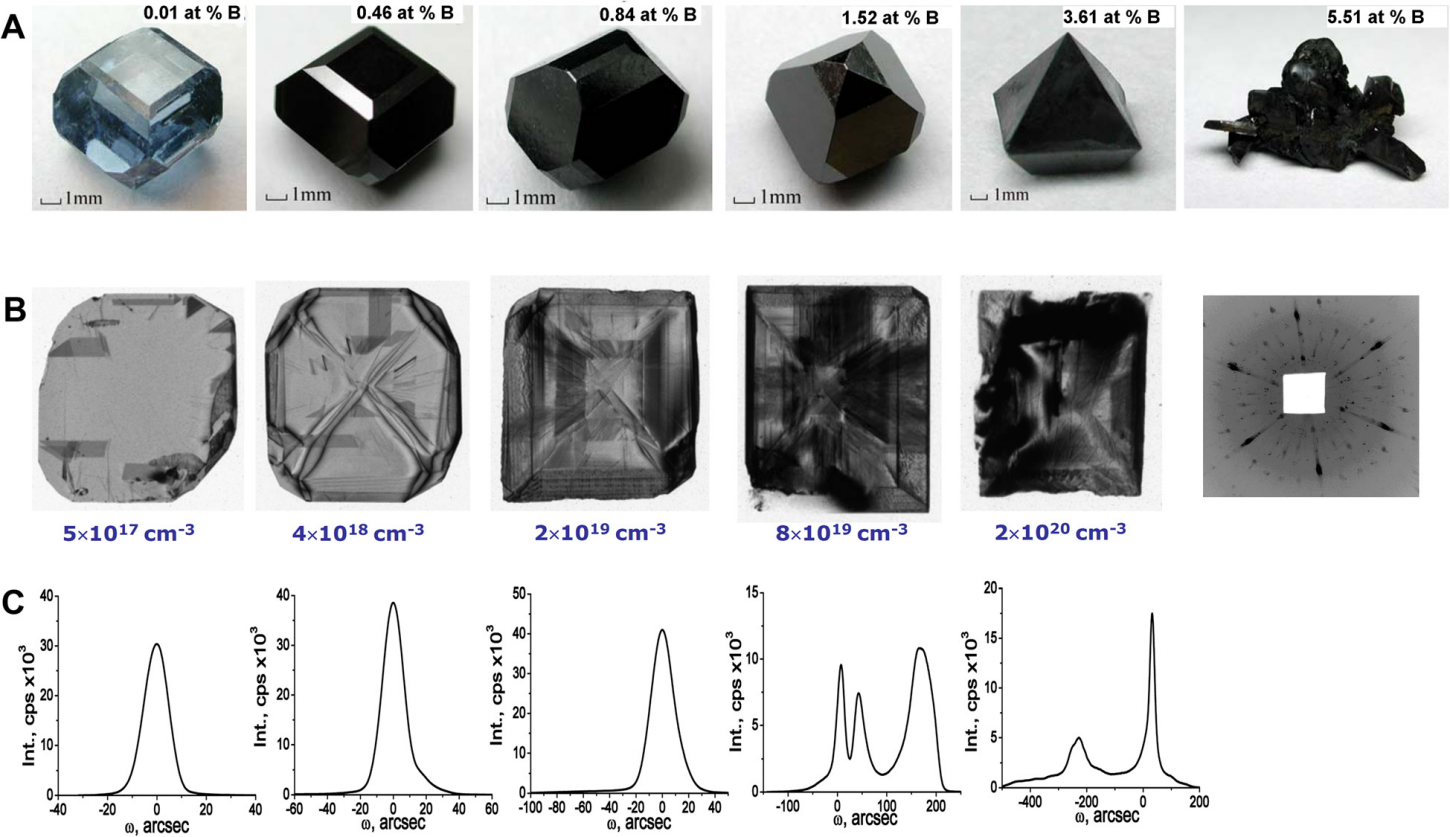
Photographs, X-ray topographs, and rocking curves of BDD. **a** Morphology of the large-sized BDD with boron content (at.% B) in the crystal growth environment. **b** X-ray traverse topographs of the (001) BDD plates with boron concentration determined by the spark mass spectrometry and the Laue pattern of (111) plate with the boron concentration of ~2 × 10^20^ cm^−3^ (*far right*). The sixfold symmetry indicates the presence of a twin. The additional small-sized spots are the satellites from a 2D incommensurately modulated structure. **c** The high-resolution double-crystal *ω*-scan rocking curves of BDD, CuKα_1_ radiation, and Si(220) channel-cut monochromator. Panels **a**–**c** from top to bottom refer to the same crystal

Figure 1a shows that the crystal morphology varies from cuboctahedron to octahedron with increasing boron concentration. The boron content in Fig. 1a corresponds to the boron concentration in the crystal growth environment. The boron content in Fig. 1b corresponds to the boron concentration in the BDD single crystal measured by mass spectrometry.

No large-sized single crystals were obtained when the boron content in the crystal growth environment exceeded ~4.0 at.% (the highest boron concentration in single crystals did not exceed ~3 × 10^20^ cm^−3^) due to twinning (Fig. 1a, far right).

**Fig. 2:**
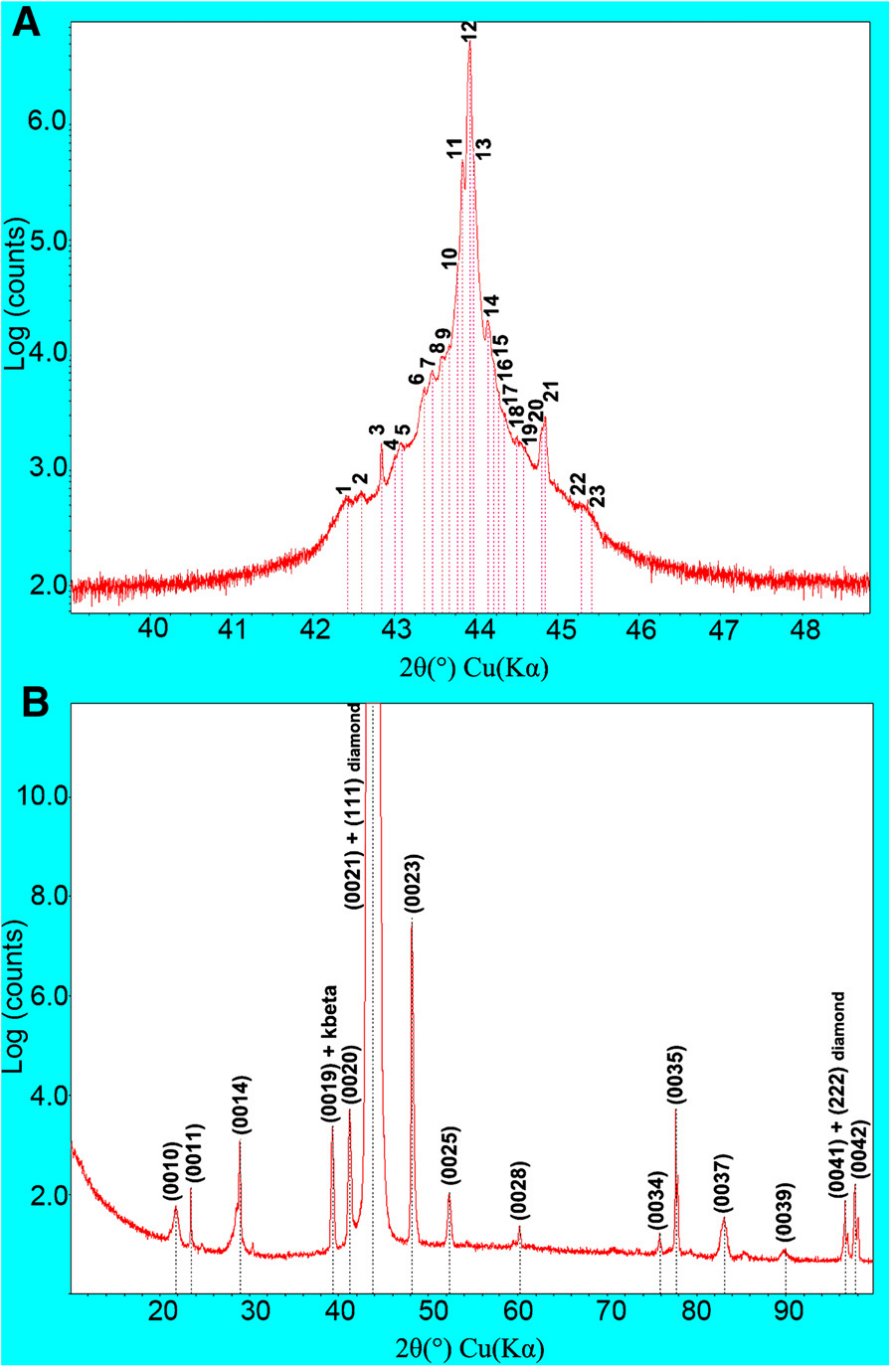
High-resolution and *θ*/2*θ*-scan X-ray patterns of the BDD with boron concentration of ~2 × 10^20^ cm^−3^. **a** X-ray double-crystal rocking curve of the (111) diamond reflection with satellites. **b** High-order diffraction peaks from 2D layer structure with a modulation period of ~43 Å in the X-ray *θ*/2*θ*-scan pattern

The X-ray topographic methods provide a picture of the defects distribution in BDD. Figure 1b shows the defect distribution in the (100) BDD plates with increase of the boron concentration. The black areas in the X-ray topographs are due to the distortion of the lattice around the defects. The mechanism of contrast is a loss of extinction when the diffraction intensity becomes proportional to the square of the structure factor. The high-resolution double-crystal X-ray diffractometry gives the quantitative information about crystal perfection based on the rocking-curve broadening analysis. The full width at half maximum of the double-crystal rocking curves of ~10 arc sec shows that BDD crystals are nearly perfect up to a boron concentration of ~2 × 10^19^ cm^−3^. The mosaic blocks (Fig. 1b, bright areas in the fourth and fifth topographs) with mutual misorientation angles up to 600 arc sec (Fig. 1c) are observed in the BDD plates above this doping level. The number of peaks on the rocking curves (three and two peaks on Fig. 1c) corresponds to the number of mosaic blocks. The help of a PANalytical hybrid Ge 4×(220) monochromator in order to increase the sensitivity for registration of the weak X-ray reflections along with a Medipix2 PIXel^3D^ detector having a very large dynamic range has allowed us to detect the weak satellite peaks in double-crystal rocking curves near the strong (111) diamond reflection (Fig. 2a, Table S1, Additional file 1). Weak high-order reflections (Fig. 2b) were recorded by means of a conventional Rigaku D/max-RC diffractometer in the step-scan mode (10 *s* per step).

The observation of the satellite and high-order reflection peaks is the “fingerprints” of a 2D incommensurate layer structure [18–22]. The average distance between B-C bilayers (modulation period) was found to be *Λ* ~ 43 Å (Fig. 2b) from the high-order diffraction peak positions according to the relation *Λ* = (*N*_i_ − N_j_) ⋅ *λ*/[2(sin*θ*_i_ − sin*θ*_j_)], where *λ* = 1.5406 Å is the X-ray wavelength, *N*_i_ and *N*_j_ are two diffraction orders, and *θ*_i_ and *θ*_j_ are the diffraction angles of these orders. These X-ray diffraction data were only obtained in BDD with the boron concentration of ~2 × 10^20^ cm^−3^. Many additional spots in the Laue pattern (Fig. 1b, far right, Figure S1 B, Additional file 1) provide the evidence of the existence of many modulation wave vectors, the directions and lengths of which do not coincide with that of the scattering vectors of the basic structure, and confirm the 2D incommensurate (misfit) layer structure of BDD with the same boron concentration. “Misfit” means that the distance between B-C layers is incommensurable with the interatomic distances of the diamond lattice. Thus, the X-ray diffraction analysis has shown that the BDD crystal at high boron concentration has the 2D misfit layer structure. The 2D layer structure in the subsurface of the same BDD was also observed in the high-resolution transmission electron microscope image [23]. The boron content in B-C layers identified by the electron energy loss spectroscopy was significantly greater than that in the diamond matrix. Note, the extensive defects like stacking faults or twins were not observed. According to the estimation [23], the boron concentration in BDD cannot exceed ~2 × 10^18^ cm^−3^ because of the high elastic strain energy of the diamond lattice which hinders the crystal growth. This argumentation motivates us to propose a model of the BDD structure.

**Fig. 3:**
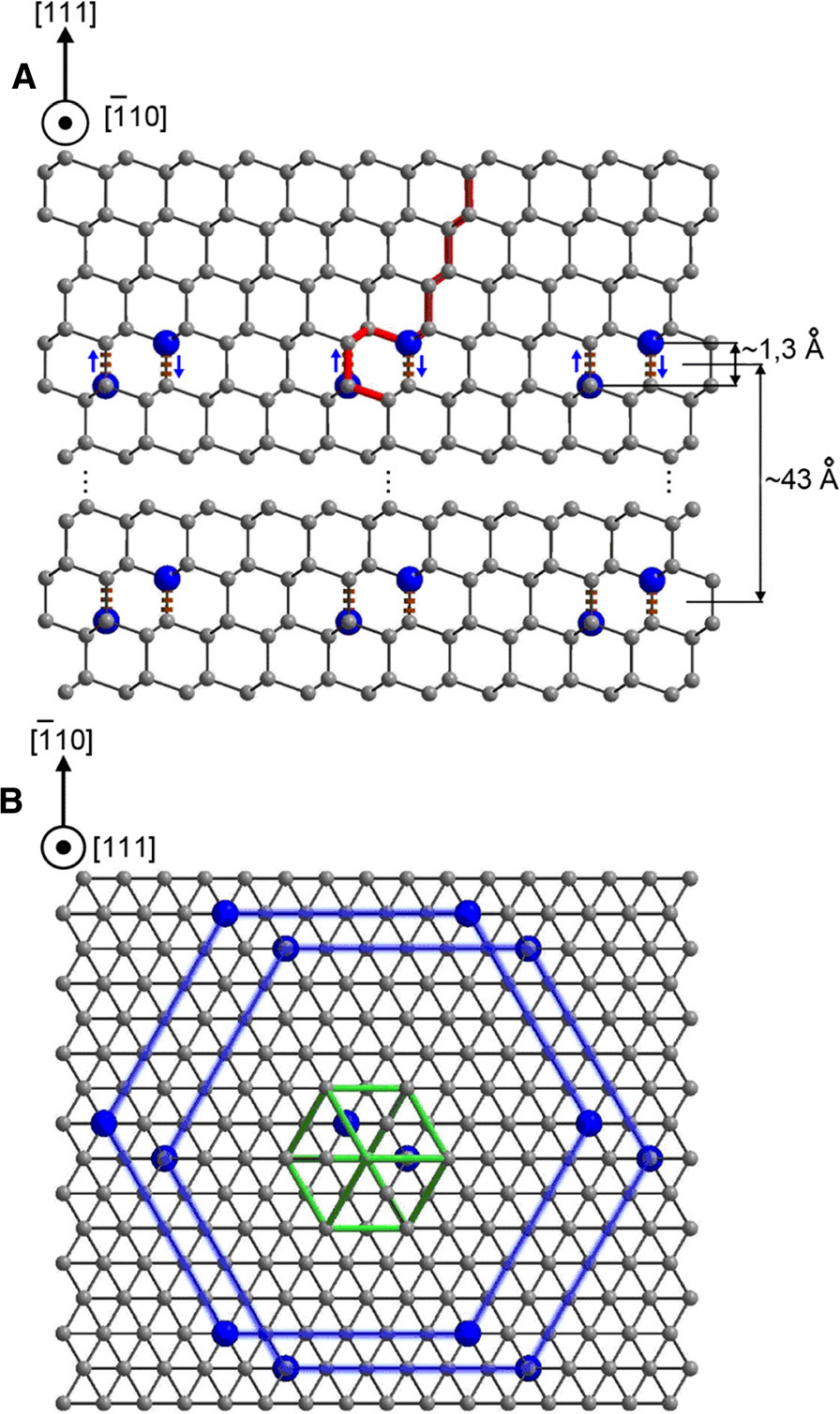
Model of the BDD structure. **a** Diagram showing a distribution of the boron (*blue*) and carbon (*gray*) atoms in the ($\overline{1}$10) plane. *Arrows* indicate the displacement of boron atoms along the [111] direction. The cubic and hexagonal stacking sequences are highlighted in *red*. The distance between boron atoms after the displacement is 1.3 Å. The average modulation period is 43 Å for a BDD crystal having a boron concentration of ~2 × 10^20^ cm^−3^. **b** Projection of 2D structure on the (111) plane

This model is based on the substitution of two carbon atoms in the (1/2, 1/2, 0) and (1/4, 3/4, 3/4) positions of the diamond unit cell by two boron atoms (Fig. 3a, b). Since the B-C bonds (1.6 Å) are longer than the C-C bonds (1.54 Å), the boron atoms are shifted towards each other along the [111] direction (Fig. 3a, marked by arrows) and their displacement provides the elastic strain relaxation. The boron pair changes the cubic ABCABC stacking sequence on the hexagonal CACA stacking sequence (red-highlighted in Fig. 3a and Figure S3, Additional file 1). A 64-atom cubic cell of diamond with the substitution of two carbon atoms by boron atoms in the positions (1/2, 1/2, 0) and (1/4, 3/4, 3/4) of the conventional cell of diamond was used for DFT simulation. It appeared that the forces 10^−4^ eV/Å in this 64-atom cell were obtained with the smaller number of the self-consistent field cycles than in the 10-atom hexagonal cell pointing out the better relaxation of the elastic strain in this cell. The boron atoms are bonded through two carbon atoms, forming a B-C-C-B chain, and no B-B dimers exist. With increasing boron concentrations, the boron pairs form double-atomic-layer-thick hexagons named the B-C nanosheets. Hexagons with borons are the most preferred on the {111} diamond planes because they have the densest structure and the same rotational symmetry as diamond. The B-C nanosheets self-assemble in the B-C bilayers at high boron concentrations (Fig. 3b). The 1.3-Å distance between boron atoms in B-C bilayers is incommensurate with that of 1.54 Å between carbon atoms of the diamond lattice in the [111] direction. We emphasize that the pair of boron atoms (in B-C-C-B chains shown at Fig. 3a) plays a key role in the formation of the 2D incommensurately modulated structure due to the displacement of those boron atoms towards each other. These displacements are responsible for the incommensurability defining the appearance of the satellite reflections and additional Laue spots (Fig. 2a and Fig. 1b, far right).

Raman scattering studies of BDD were used to complement the X-ray diffraction data, since this technique is highly sensitive to impurity-related defects, which enable investigations of the boron incorporation in the diamond lattice in the wide range of boron concentrations while the X-ray diffraction method is insensitive to either point defects or nanosheets. The highly penetrating X-rays (with the penetration depth of ~0.6 mm) provide the structural information on the bulk of BDD, while resonant (514-nm laser wavelength) and nonresonant (257-nm laser wavelength) Raman scattering can probe the surface layers, the bulk crystal, or combinations thereof. The relations of the absorption coefficients in BDD for 514- and 257-nm laser wavelengths are *α*_514_/*α*_257_ ≈ 2 ÷ 3 at the low boron concentration [24], *α*_514_/*α*_257_ ≈ 5 ÷ 8 at the middle boron concentration, and *α*_514_/*α*_257_ ≈ 20 ÷ 25 at the high boron concentration.

**Fig. 4:**
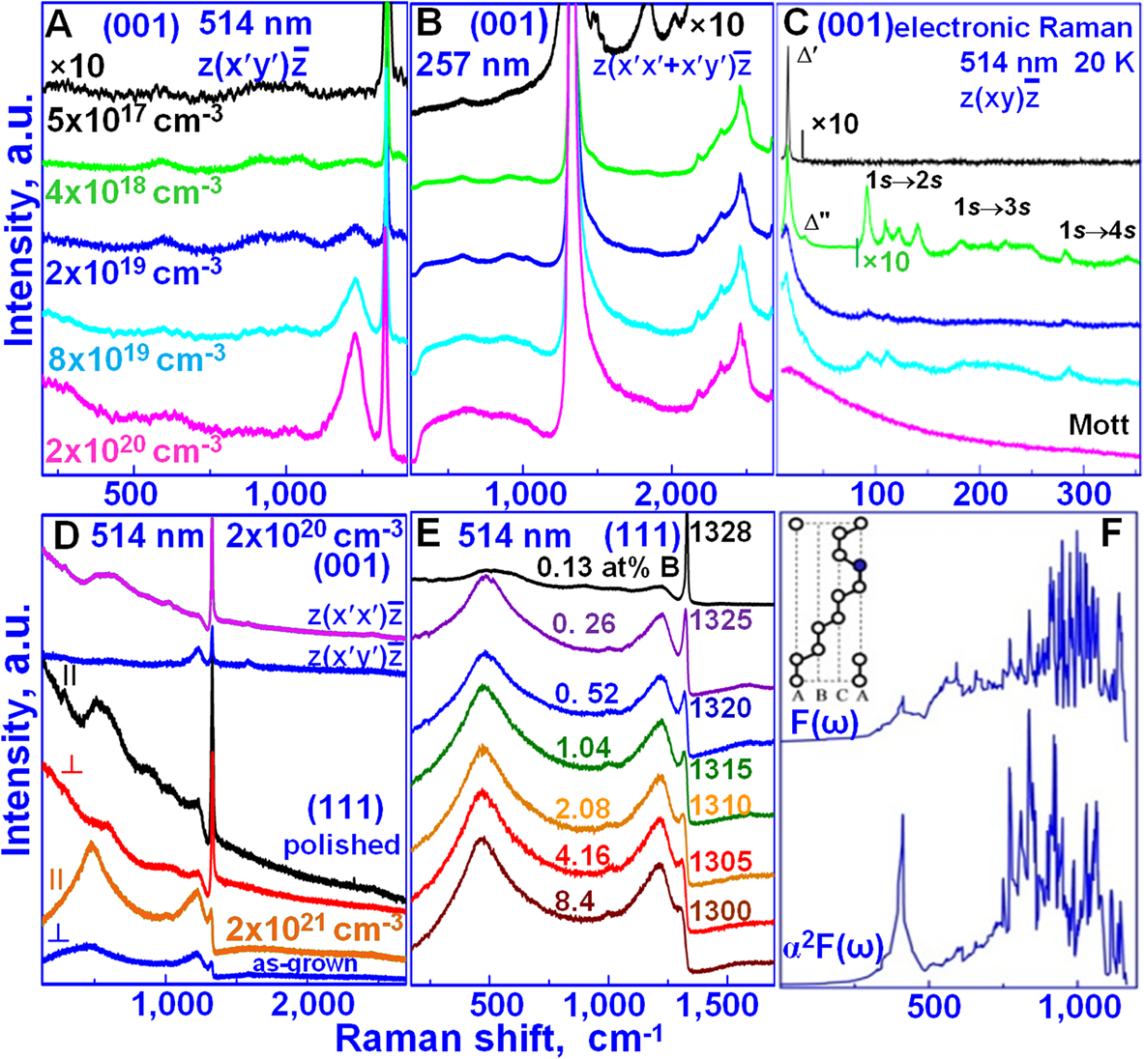
Vibrational and electronic Raman spectra of BDD. **a** Evolution of the Raman spectra of the (001) BDD plates with increasing boron concentration in backscattering along z||[001]. The incident light is polarized along x′||[110], and the scattered light is analyzed along y′||[1 $\overline{1}$ 0]. The spectra were excited with the 514-nm laser line. **b** Evolution of the unpolarized Raman spectra of the same (001) BDD plates excited with the 257-nm laser line. **c** Evolution of the electronic Raman spectra of the same (001) BDD plates excited with the 514-nm laser line, recorded at 20 K in the backscattering geometry. The same colors in **a**–**c** correspond to BDD plates with the same boron concentration. **d** Raman spectra of the as-grown and polished (001) and (111) surfaces of the BDD in different backscattering geometries excited with the 514-nm laser line. **e** The dependence of the resonant Raman spectra from the (111) surface of BDD on the boron concentration and on the modulation period from 43.25 Å (*top*) to 6.18 Å (*bottom*) with a step of 6.18 Å. **f** The phonon density of states *F*(*ω*) and the Eliashberg phonon spectral function *α* ^2^ *F*(*ω*) calculated for the ABCACA sequence shown in the inset

The evolution of the Raman spectra of BDD with increasing boron concentration is shown in Fig. 4a, b for excitation at the 514- and 257-nm laser wavelengths, respectively. Fig. 4a, b are normalized to the integral intensity of the 1332 cm^−1^ diamond peak (and, correspondingly, to the intensity of its second order) to take into account the decrease of the scattering volume with increasing boron concentration.

**Fig. 5:**
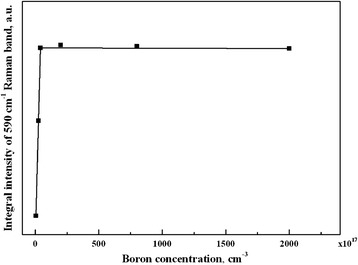
Dependence of the integral intensity of the 590-cm^−1^ defect-induced band excited with the 257-nm laser on boron concentration

At low boron concentrations up to ~10^18^ cm^−3^, the boron substitutes carbon in the diamond unit cell keeping the O_h_ symmetry of defects (see Additional file 1), but the inversion symmetry at this site and the *q* = 0 wave vector selection rule is broken. The resulting Raman spectra are proportional to the weighted PDOS of the perturbed diamond structure. The wide bands at 588, 890, and 1042 cm^−1^ are the defect-induced first-order Raman bands reflecting the PDOS in the perturbed structure of BDD (Fig. 4a, b and Figure S2, Additional file 1). As seen from these spectra, their intensities increase almost linearly with increasing boron concentration from 5 × 10^17^ cm^−3^ up to 4 × 10^18^ cm^−3^ and do not practically change from 4 × 10^18^ cm^−3^ up to 2 × 10^20^ cm^−3^ (Fig. 5).

This means that the boron atoms are not incorporated into the diamond matrix at a doping level above ~4 × 10^18^ cm^−3^. This is a reason why the diamond lattice constant in this range of boron concentrations does not change as was observed [11]. At boron concentrations above ~4 × 10^18^ cm^−3^, a pair of borons that substitutes two carbons in a diamond unit cell (as proposed in our structure model of BDD) changes the cubic O_h_ symmetry of the substitutional boron defect on the hexagonal D_6h_ symmetry of the substitutional boron pair defect. In fact, a new broad Raman band at 1230 cm^−1^ arises in the $z\left(x^{\prime }y^{\prime}\right)\overline{z}$ backscattering geometry (green spectrum in Fig. 4a), and its intensity increases almost linearly with increasing doping level (Fig. 4a). Another new intense broad Raman band at 480 cm^−1^ appears together with the band at 1230 cm^−1^ in the $z\left(x^{\prime }x^{\prime}\right)\overline{z}$ backscattering geometry (magenta spectrum in Fig. 4d). The polarization properties of these new Raman bands indicate their D_6h_ symmetry (see Additional file 1). We assign the bands at 480 and 1230 cm^−1^, respectively, to transverse acoustic and optical branches of PDOS of the B-C nanosheets. These bands are only seen in Raman spectra excited with the 514-nm laser wavelength because of the resonant character of Raman scattering caused by absorption of this laser line in the B-C nanosheets which are nearly transparent for the 257-nm laser wavelength [24]. It is namely the absorption of visible light in the B-C nanosheets that leads to the black color of BDD. DFT calculations of the vibrational properties based on the proposed structural model of BDD with the basis containing the ABCACA stacking sequence (red-highlighted in Fig. 3a, the inset in Fig. 4f, and Figure S3, Additional file 1) were carried out using Quantum Espresso package (http://www.quantum-espresso.org). Calculations were performed for the maximum boron concentration of 8.4 at.% which can be reached in the (111) BDD surface (Fig. 4e, bottom spectrum). The outcome of the DFT calculation (Fig. 4f) is the appearance of bands at ~410 and ~1100 cm^−1^, which reflect the transverse acoustic and the optical branches of the PDOS (*F*(*ω*)) of B-C bilayers. Since the Raman scattering in B-C bilayers has a resonant character, the intensities of these bands will be proportional to the square of the electron-phonon coupling *α* and can be approximated by the *α*^2^*F*(*ω*) Eliashberg spectral function (Fig. 4e). This function shows that the electron-phonon coupling is high for transverse acoustic vibration. Therefore, the intensities of the 480 and 1230 cm^−1^ bands observed experimentally are comparable. We have evidently shown that the Raman spectra of BDD are indeed the superposition of spectra obtained from the B-C bilayers and the bulk diamond matrix. We underline that the boron atoms are mainly incorporated in the B-C nanosheets and bilayers once the doping level exceeds the equilibrium concentration of boron atoms in the bulk diamond lattice, namely starting with boron concentrations of ~4 × 10^18^ cm^−3^.

**Fig. 6:**
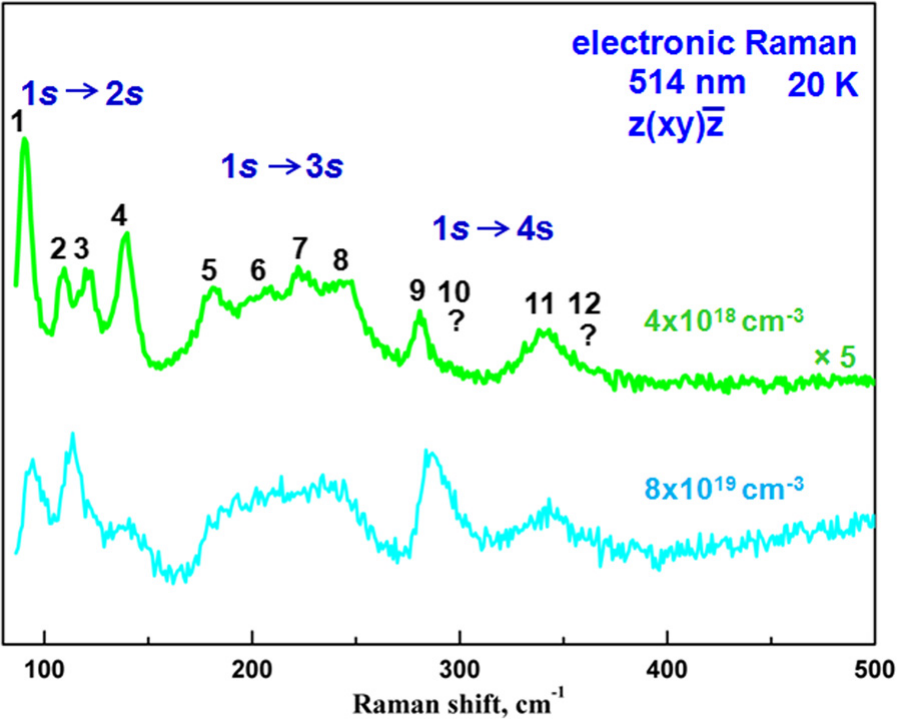
Electronic Raman spectra of BDD in the wavenumber range of 90–500 cm^−1^

The B-C nanosheet formation should affect the electronic structure of the acceptor states of BDD that we observe in our experiments. Figure 4c and Fig. 6 show the evolution of the electronic Raman spectra of BDD with increasing doping level. Figure 6 is given to see more clearly the intensity changes of the electronic Raman spectra in the larger wave number range.

**Fig. 7:**
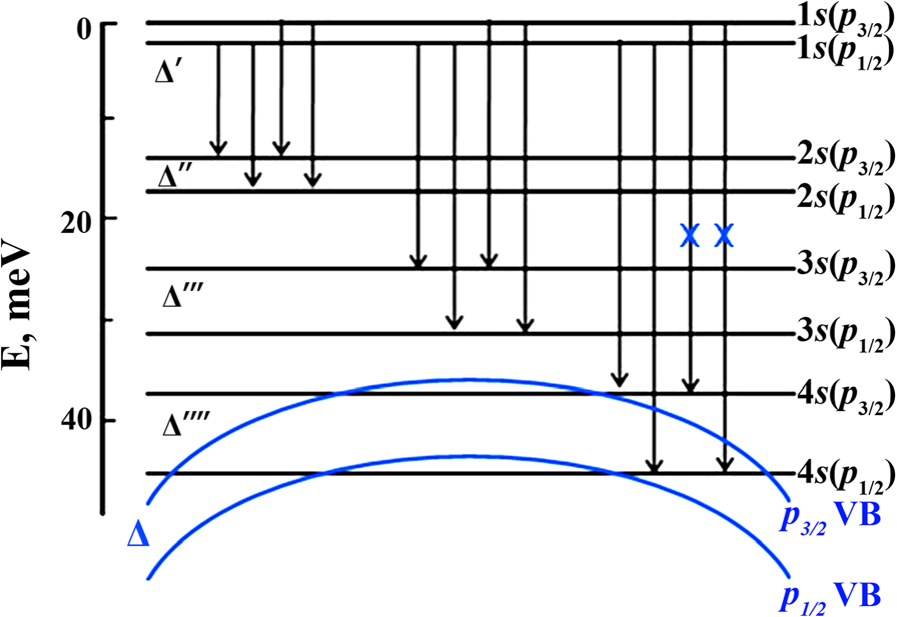
The energy-level scheme of boron acceptor states in BDD with the Δ, Δ′–Δ′′′′ spin-orbit splitting of the valence band and the n*s* acceptor states and electronic transitions from the ground state to the n*s* excited states

At low boron concentrations up to ~10^18^ cm^−3^, the Raman band at *∆*′ = 16 cm^−1^ assigned to the 1*s*(*p*_3/2_) → 1*s*(*p*_1/2_) electronic transition between the component of the spin-orbit splitting of the 1*s* ground acceptor state of BDD [25] is only observed. After the B-C nanosheet formation at the boron concentration of ~4 × 10^18^ cm^−3^, we start to see the 1*s* → n*s* Lyman series of electronic transitions between the 1*s* ground and the n*s* excited acceptor states, separated by about 13 meV, with four bands each because of the spin-orbit splitting of the ground and excited acceptor states. The spin-orbit splitting of the n*s* acceptor states is increased linearly with the *n* principle quantum number [26]. In this case, the wave functions of acceptor states begin to overlap populating the n*s* exited states but remain isolated and localized. Consequently, the bands of electronic transitions are narrow. Frequencies and energies of the *Δ*′–*Δ*′′′′ spin-orbit splitting of the n*s* boron acceptor states and Lyman electronic transitions from the 1*s*(*p*_3/2_) and 1*s*(*p*_1/2_) ground states to the n*s*(*p*_3/2_) and n*s*(*p*_1/2_) excited states in the BDD with the boron concentration of ~4 × 10^18^ cm^−3^ obtained from the electronic Raman spectra (Fig. 4c and Fig. 6, green spectra) are given in Table S3 (Additional file 1). The observation of only two bands in the 1*s* → 4*s* electronic transitions instead of four bands in the 1*s* → 2*s* and 1*s* → 3*s* electronic transitions and the absence of the 1*s* → 5*s* transitions (Fig. 4c and Fig. 6) demand an explanation. The energy-level scheme of boron acceptor states in BDD with the *∆*, *∆*′–*∆*′′′′ spin-orbit splitting of valence band and the n*s* acceptor states and electronic transitions from the ground state to the n*s* excited states is presented in Fig. 7. This energy-level scheme is needed for an analysis of the electronic Raman spectra.

The absence of the 1*s*(*p*_3/2_) → 4*s*(*p*_3/2_) electronic transition (10? in Fig. 6 and Table S3, Additional file 1; marked blue cross in Fig. 7) caused by merging the *p*_3/2_ valence band (upper blue line in Fig. 7) with the 4*s*(*p*_3/2_) boron acceptor state indicates unambiguously on the formation of a new shallow acceptor level at ~37 meV associated with B-C nanosheets. The dark conductivity with the activation energy of 22 meV in BDD [13] may be associated with this acceptor level. On the other hand, the absence of the 1*s*(*p*_3/2_) → 4*s*(*p*_1/2_) electronic transition (12? in Fig. 6 and Table S3, Additional file 1; marked blue cross in Fig. 7) caused by merging the *p*_1/2_ valence band (lower blue line in Fig. 7) with the 4*s*(*p*_1/2_) boron acceptor state and broadening of the electronic Raman band at 345 cm^−1^ (11 in Fig. 6 and Table S3, Additional file 1) assigned to the 1*s*(*p*_1/2_) → 4*s*(*p*_1/2_) electronic transition determine the energy level of the *p*_1/2_ valence band of ~43 meV and the spin-orbit splitting of the valence band (*∆*) of ~6 meV which is in good agreement with the *∆* value measured by Rauch [27]. With the boron concentration increase, the bands of electronic transitions are broadened because of the delocalization of the acceptor states and their intensities are increased. Finally, in the formed B-C bilayers at the boron concentration of 2 × 10^20^ cm^−3^, the Mott transition occurs when the acceptor states merge with the top of the valence band and the 1*s* → n*s* electronic transitions are not already observed in the electronic Raman spectrum (Fig. 4c, bottom spectrum). Thus, the electronic Raman spectra exhibit the electronic transitions between the acceptor states which are associated with the B-C nanosheets, the new shallow acceptor level at ~37 meV, the spin-orbit splitting of the valence band of ~6 meV, and the Mott transition in the B-C bilayers.

The resonant Raman spectra from the as-grown and polished (111) BDD surfaces differ drastically (Fig. 4d). The origin of this difference is explained by the fact that boron content in the as-grown surface is approximately 10 times higher than that in the bulk, as was experimentally determined [28]. Indeed, the Raman spectra of BDD are the superposition of spectra obtained from the surface and the bulk diamond matrix. The bands of the boron bilayers prevail in the Raman spectrum of the as-grown surface (Fig. 4d, orange spectrum). In contrast, both the bands of the B-C bilayers and these of the diamond matrix are observed in the Raman spectrum of the same BDD plate with the boron concentration of ~2 × 10^20^ cm^−3^ as in the bulk after polishing the as-grown surface (Fig. 4d, black spectrum).

Figure 4e shows the dependence of the resonant Raman spectra from the (111) surface of BDD on the boron concentrations estimated from the proposed structure model (see Additional file 1.4). Again, we see the superposition of Raman spectra from the diamond matrix and more intense spectra from the B-C bilayers. Note that the (111) surfaces of the as-grown BDD crystal are strongly inhomogeneous. The degree of inhomogeneity is so large that the position of the diamond peak is varied in the range of 1328–1300 cm^−1^ from the different regions of the same surface. We found that these diamond peak shifts with the step of ~5 cm^−1^ and its intensity correspondingly decreases (Fig. 4e). The origin of this step-like shift can be explained by our structure model. The number of boron atoms in the boron bilayers and the distance between these bilayers (modulation period, shown as 43 Å in Fig. 3) determine the total boron concentration (see Additional file 1). The modulation period corresponds to multiple interplanar distances of 2.06 Å in the [111] direction of the diamond. It changes from 43 Å within the bulk as determined by X-ray up to 6.18 Å (the length of the main diagonal of the diamond unit cell) on the surface. The stepwise reduction of the modulation period leads to the intensity decrease of diamond peak and to the step-like shift of this peak associated with the 1D phonon confinement along [111] direction in the thin diamond layers.

We identified that the transitions to the superconducting state occur in the (111) surfaces of the as-grown BDD crystal only [29]. We found that the shift of the diamond peak correlates with a temperature of superconducting transition *T*_c_. For the detected superconducting transitions at temperatures *T*_c_ ≈ 2 K and *T*_c_ ≈ 4 K, the positions of diamond peaks were 1310 and 1305 cm^−1^, respectively. We cannot detect the superconductivity in the surface areas where the positions of diamond peaks were ≥1315 cm^−1^. Hence, the position of diamond peak can serve as indicator for detection of superconducting areas on the surface. We assume that the superconductivity in the BDD is associated with alternating metallic B-C bilayers and the semiconducting diamond layers, in accordance with the Ginzburg suggestion [17]. The smaller distance between B-C bilayers leads to higher *T*_c_.

## Conclusions

In summary, we have demonstrated the formation of B-C nanosheets and bilayers in BDD with increasing boron concentration that result in the elastic strain relaxation of the diamond matrix. Namely, the B-C nanosheets give rise to the appearance of Raman bands at 480 and 1230 cm^−1^ and the observation of the 1*s* → n*s* electron-acceptor transitions in the electronic Raman spectra. The new shallow boron acceptor level at ~37 meV and the spin-orbit splitting of the valence band of ~6 meV were defined from the analysis of the electronic Raman spectra of BDD. The B-C nanosheets self-assemble in the B-C bilayers at high boron concentration (~2 × 10^20^ cm^−3^) in BDD. The evidence for these B-C bilayers which are parallel to {111} planes is provided by the observation of the high-order and satellite reflections in X-ray diffraction and the additional spots in Laue patterns. From X-ray data, we conclude that the BDD with a high boron concentration has the 2D misfit layer structure with a modulation period of ~43 Å. We propose the model for the BDD crystal structure which is based on X-ray and Raman data and confirmed by DFT calculation. The Mott transition which is observed in electronic Raman spectra occurs within the bulk BDD and is associated with the metallic B-C bilayers. The superconducting transitions were only detected on the surface of the overdoped BDD having a short modulation period between the B-C bilayers. Knowledge of the nanostructure in BDD will help in designing diamond electronics and understanding the nature of the metallic conductivity and superconductivity for other elements of the fourth group of the periodic table in which these phenomena may exist. The alternating B-C bilayers in BDD with modulation periods of 6.18–43 Å may be considered as multilayer mirrors and Bragg and Laue X-ray interferometers. The design of these interferometers for soft and hard X-rays has drawn significant interest for synchrotron applications.

## Additional file

Additional file 1:
**Supplementary information.** (PDF 603 kb)

## Abbreviations

1D, 2D, and 3D: one-, two-, and three-dimensional
B: boron atoms
BDD: boron-doped diamond
C: carbon atoms
DFT: density functional theory
PDOS: phonon density of states
