# Supplementary material for: Formation of Boron-Carbon Nanosheets and Bilayers in Boron-Doped Diamond: Origin of Metallicity and Superconductivity
Source: Nanoscale Res Lett. 2016 Jan 12;11:11. doi: 10.1186/s11671-015-1215-6 (PMC4709361; doi:10.1186/s11671-015-1215-6)
Supplement: Additional file 1: — Supplementary information. (PDF 603 kb) [file 11671_2015_1215_MOESM1_ESM.pdf]

Additional file 1: Supplementary information for

Formation of boron-carbon nanosheets and bilayers in boron doped diamond: origin of metallicity and superconductivity

SN Polyakov[1*], **VN Denisov**[1 2*], **BN Mavrin**[2], **AN Kirichenko**[1], **MS Kuznetsov**[1], **SYu Martyushov**[1], **SA Terentiev**[1] and **VD Blank**[1]

[1] *Technological Institute for Superhard and Novel Carbon Materials, Troitsk, Moscow, 142190, Russia.*

[2] *Institute of Spectroscopy, Russian Academy of Sciences, Troitsk, Moscow, 142190, Russia.*

[*]Corresponding Authors: spolyakov@phys.msu.ru (SN Polyakov), denisovvn@tisnum.ru (VN Denisov)

Overview of the Supplementary information

1. Observation of 2D layer structure by X-ray

2. Selection rules for Raman scattering in BDD

3. Electronic Raman scattering in BDD

4. Simple calculation of boron content from the structure model

1. Observation of 2D layer structure by X-ray

The presence of weak satellite peaks in the double-crystal rocking curve near the strong (111) diamond reflection (Fig. 2A, main paper) and the high-order diffraction peaks in the θ/2θ-scan

#	Bragg angle 2θ(^{0})	d – Spacing (Å)	Height (Counts)	Δd (Å)	Scattering vector q_i = 21×d (Å)
1	42.424	2.1290	436	0.0696	44.7090
2	42.594	2.1209	496	0.0615	44.5389
3	42.844	2.1090	1573	0.0496	44.2890
4	43.002	2.1017	1102	0.0423	44.1357
5	43.090	2.0976	1516	0.0382	44.0496
6	43.366	2.0849	4606	0.0255	43.7829
7	43.466	2.0803	6764	0.0209	43.6843
8	43.582	2.0750	9131	0.0156	43.5750
9	43.670	2.0711	11320	0.0117	43.4931
10	43.776	2.0663	54295	0.0069	43.3923
11	43.836	2.0636	497208	0.0042	43.3356
**12**	**43.930**	**2.0594**	**5324710**	**0.0000**	**43.2474**
13	43.972	2.0575	581389	0.0019	43.2075
14	44.144	2.0499	19540	0.0095	43.0479
15	44.218	2.0467	8303	0.0127	42.9807
16	44.274	2.0442	4275	0.0152	42.9282
17	44.346	2.0410	2656	0.0184	42.8610
18	44.498	2.0344	1612	0.0250	42.7224
19	44.580	2.0309	1356	0.0285	42.6489
20	44.806	2.0211	2129	0.0383	42.4431
21	44.850	2.0193	2749	0.0401	42.4053
22	45.290	2.0007	363	0.0587	42.0147
23	45.416	1.9954	266	0.0640	41.9034

Table S1 Positions of the satellite peaks on the X-ray double-crystal rocking curve of the (111) boron doped diamond plate with the boron content of ~2×10^{20} cm^{-3}. Last column indicates the length of the scattering vectors q_i.

pattern (Fig. 2B, main paper) are the "fingerprints" of a 2D layer structure. The satellite peak reflections near the (111) double-crystal rocking curve of diamond are associated with the incommensurate modulations in the [111] direction of diamond lattice with independent q_i vectors. Table S1 presents the satellite peak positions on Fig. 2A. The average modulation period $\Lambda \sim 43$ Å is determined from the high-order diffraction peaks position on Fig. 2B according to the relation $\Lambda = (N_i - N_j)\cdot\lambda/[2(\sin\theta_i - \sin\theta_j)]$, where $\lambda = 1.5406$ Å is X-ray wavelength, N_i and N_j are two diffraction order and θ_i and θ_j are diffraction angles of these orders.

The appearance of a new spots on the Laue pattern of the heavily BDD is an additional confirmation that BDD has the 2D incommensurately modulated structure (see Fig. S1).

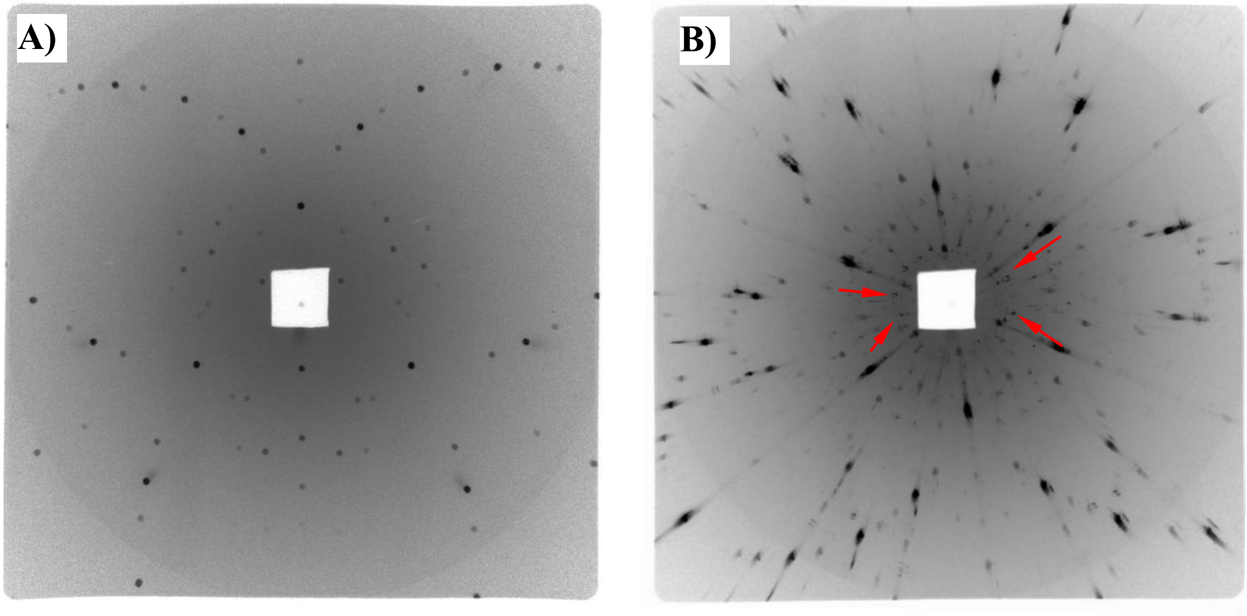

Fig. S1 A) Laue pattern of the BDD with boron concentration of 2×10^{19} cm^{-3}. The additional spots are absent. **B)** Laue pattern of (111) BDD plate with the boron concentration $\geq 2\times10^{20}$cm^{-3}. The additional small-sized spots some of which indicated by arrows are due to the 2D incommensurately modulated structure.

2. Selection rules for Raman scattering in BDD

Diamond belongs to the space group O_h^7 with two atoms per primitive cell leading to three acoustic (F_{1u}) and three optical (F_{2g}) branches in the phonon-dispersion picture of the Brillouin zone (Fig. S1 E) [1]. The F_{2g} triply degenerated mode of 1332 cm^{-1} at the center of the zone (Γ) is Raman active. When a single boron atom substitutes a carbon one at doping level up to 4×10^{18} cm^{-3} the cubic diamond structure is perturbed and the q=0 wave vector selection rule is broken. The point defects induce Raman activity of singularities of the phonon density of states (PDOS) near the zone boundary [2, 3] (Fig. S2). The Raman tensors will have the O_h symmetry properties with the irreducible nonzero components for A_{1g}, E_g and F_{2g} phonon modes. The Raman selection rules for diamond in the backscattering geometries shown in Fig. S2 for (001) and (111) plains are given in Table S2 (following Ref. [4]). As seen from Fig. S2, the 588 and 1042 cm^{-1} Raman bands of the F_{2g} symmetry correspond to transverse (TA) and longitudinal (LA) acoustic phonon branches and reflect the singularities in the PDOS of diamond. The 890 cm^{-1} Raman band of the A_{1g} symmetry can be assigned to the resonance-exited mode (RM) which is activated by weakly bound boron atoms in diamond lattice [5].

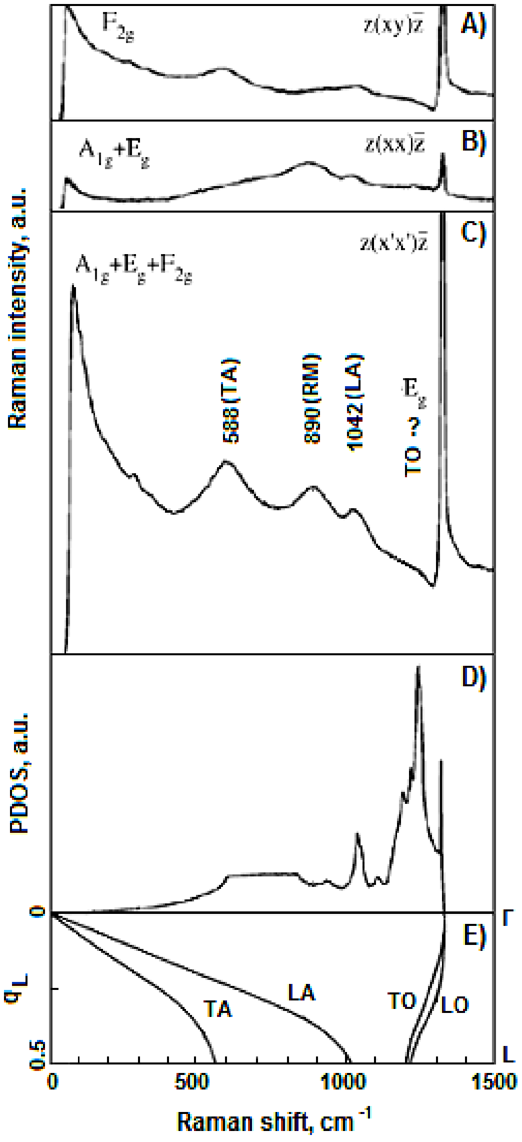

Fig. S2 A-C) Polarized Raman spectra of BDD with boron concentration of 4×10^{18}cm^{-3}, **D)** Phonon density of states of diamond, **E)** Phonon-dispersion curves of diamond along the Λ direction in the Brillouin zone, where $q_L = (2\pi/a)$ (1/2,1/2,1/2) represents the phonon wave vector and a is the lattice constant.

Plain	Laser light polarization	Scattered light polarization	Raman activity of phonon modes
(001)	[100]	[010]	F_{2g}
(001)	[100]	[100]	$A_{1g} + E_g$
(001)	[110]	[110]	$A_{1g} + E_g + F_{2g}$
(001)	[110]	$[1\bar{1}0]$	E_g
(111)	$[1\bar{1}0]$	$[1\bar{1}0]$	$A_{1g} + E_g + F_{2g}$
(111)	$[1\bar{1}0]$	$[11\bar{2}]$	F_{2g}

Table S2 Raman selection rules for diamond.

The polarization properties of Raman spectra of BDD at low boron doping level are in good agreement with selection rules for the O_h point group. Note, that TO and LO optical phonon branches do not reveal in Raman spectra of BDD. Only the E_g phonon mode might be Raman active in the $z(x'y')\bar{z}$ backscattering geometry. The appearance of the 588 cm^{-1} (TA), 890 cm^{-1} (RM) and 1042 cm^{-1} (LA) Raman bands of the A_{1g} and F_{2g} symmetries in this geometry (Fig. 4A, main paper) is due to the 7% breakthrough from the $z(x'x')\bar{z}$ one because of the large collection angle of the scattered light and allows us to normalize the Raman spectra to the integral intensity of the 1332 cm^{-1} diamond peak. The appearance of a new band at 1230 cm^{-1} in Raman spectra of BDD and the linear increase of its intensity with increasing doping level from ~4×10^{18} cm^{-3} up to ~2×10^{20} cm^{-3}, the absence of this band as well as a 480 cm^{-1} band at doping level lesser ~10^{18} cm^{-3} (Fig. 4A, main paper) and in the UV Raman spectra of BDD at all boron concentrations (Fig. 4B, main paper) indicate unambiguously on a formation of a boron nanosheets associated with incorporation of two boron atoms in the diamond unit cell. Fig. S3 shows that the stacking sequence in cubic diamond along the [111] direction is ABC while that in hexagonal boron nanosheets along the [0001] direction is CACA, i.e. $(111)_{cub} \parallel (0001)_{hex}$. The symmetry group of boron nanosheets is the D^4_{6h} space group.

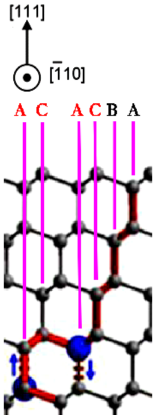

Fig. S3 Cubic and hexagonal stacking sequences in the BDD structure.

The Raman tensors will have the D_{6h} symmetry properties with the irreducible nonzero components for A_{1g}, $E_{1g}^{1,2}$ and $E_{2g}^{1,2}$ phonon modes.

To obtain the Raman selection rules for hexagonal boron nanosheets in the cubic crystallographic axes we should transform the Raman tensors of the A_{1g}, $E_{1g}^{1,2}$ and $E_{2g}^{1,2}$ phonon modes in the hexagonal crystallographic axes:

$$\mathbf{A}_{1g}=\begin{vmatrix}\mathbf{a} & 0 & 0\\ 0 & \mathbf{a} & 0\\ 0 & 0 & \mathbf{b}\end{vmatrix},\quad \mathbf{E}_{1g}^{1}=\begin{vmatrix}0 & 0 & 0\\ 0 & 0 & \mathbf{c}\\ 0 & \mathbf{c} & 0\end{vmatrix},\quad \mathbf{E}_{1g}^{2}=\begin{vmatrix}0 & 0 & -\mathbf{c}\\ 0 & 0 & 0\\ -\mathbf{c} & 0 & 0\end{vmatrix},\quad \mathbf{E}_{2g}^{1}=\begin{vmatrix}0 & \mathbf{d} & 0\\ -\mathbf{d} & 0 & 0\\ 0 & 0 & 0\end{vmatrix},\quad \mathbf{E}_{2g}^{2}=\begin{vmatrix}\mathbf{d} & 0 & 0\\ 0 & -\mathbf{d} & 0\\ 0 & 0 & 0\end{vmatrix}$$

(S1)

to transform these in cubic crystallographic axes we use following formula

$$\alpha_{\mathbf{ij}}^{\mathbf{cub}}=\rho_{\mathbf{ik}}\,\rho_{\mathbf{jl}}\,\alpha_{\mathbf{kl}}^{\mathbf{hex}}, \tag{S2}$$

where $\rho_{\mathbf{ik}}$ is the tensor of transformation from hexagonal to cubic crystallographic axes

$$\rho_{\mathbf{ik}}=\frac{1}{6}\begin{vmatrix}3 & 1 & 2\\ -3 & 1 & 2\\ 0 & -2 & 2\end{vmatrix} \tag{S3}$$

Applying formulas (S2, 3) for tensors (S1), we get the Raman tensors of the A_{1g}, $E_{1g}^{1,2}$ and $E_{2g}^{1,2}$ phonon modes in the cubic crystallographic axes with z || [111]:

$$\mathbf{A}_{1g}=\begin{vmatrix}\mathbf{a} & \mathbf{c} & \mathbf{d}\\ \mathbf{c} & \mathbf{a} & \mathbf{d}\\ \mathbf{d} & \mathbf{d} & \mathbf{b}\end{vmatrix},\ \mathbf{E}_{1g}^{1}=\frac{1}{18}\begin{vmatrix}2\mathbf{c} & 2\mathbf{c} & -\mathbf{c}\\ 2\mathbf{c} & 2\mathbf{c} & -\mathbf{c}\\ -\mathbf{c} & -\mathbf{c} & -4\mathbf{c}\end{vmatrix},\ \mathbf{E}_{1g}^{2}=\frac{1}{6}\begin{vmatrix}-2\mathbf{c} & 0 & -\mathbf{c}\\ 0 & -2\mathbf{c} & \mathbf{c}\\ -\mathbf{c} & \mathbf{c} & 0\end{vmatrix},\ \mathbf{E}_{2g}^{1}=\frac{1}{6}\begin{vmatrix}\mathbf{d} & 0 & -\mathbf{d}\\ 0 & -\mathbf{d} & \mathbf{d}\\ -\mathbf{d} & \mathbf{d} & 0\end{vmatrix},$$

$$\mathbf{E}_{2g}^{2}=\frac{1}{18}\begin{vmatrix}4\mathbf{d} & -5\mathbf{d} & \mathbf{d}\\ -5\mathbf{d} & 4\mathbf{d} & \mathbf{d}\\ \mathbf{d} & \mathbf{d} & -2\mathbf{d}\end{vmatrix} \tag{S4}$$

The Raman tensors of the A_{1g}, $E_{1g}^{1,2}$ and $E_{2g}^{1,2}$ phonon modes in the cubic crystallographic x', y', z axes, calculated after the 45° rotation around z || [001] with use of formulas (S5, 6)

$$\boldsymbol{\alpha}'_{\mathbf{ij}} = \Re_{\mathbf{ik}}\, \Re_{\mathbf{jl}}\, \boldsymbol{\alpha}_{\mathbf{kl}} \tag{S5}$$

$$\Re_{\mathbf{ik}} = \begin{vmatrix} \sqrt{2}/2 & \sqrt{2}/2 & 0 \\ -\sqrt{2}/2 & \sqrt{2}/2 & 0 \\ 0 & 0 & 1 \end{vmatrix}, \tag{S6}$$

have following representations:

$$\mathbf{A}_{1g} = \frac{1}{18}\begin{vmatrix} \mathbf{a}+4\mathbf{b} & 0 & \sqrt{2}(-\mathbf{a}+2\mathbf{b}) \\ 0 & 4\mathbf{a}-2\mathbf{b} & 0 \\ \sqrt{2}(-\mathbf{a}+2\mathbf{b}) & 0 & 2\mathbf{a}+2\mathbf{b} \end{vmatrix}, \quad \mathbf{E}^{1}_{1g} = \frac{1}{18}\begin{vmatrix} 4\mathbf{c} & 0 & -\sqrt{2}\mathbf{c} \\ 0 & -2\mathbf{c} & 0 \\ -\sqrt{2}\mathbf{c} & 0 & -4\mathbf{c} \end{vmatrix},$$

$$\mathbf{E}^{2}_{1g} = \frac{1}{6}\begin{vmatrix} 0 & 2\mathbf{c} & 0 \\ 2\mathbf{c} & 2\mathbf{c} & \sqrt{2}\mathbf{c} \\ 0 & \sqrt{2}\mathbf{c} & 0 \end{vmatrix}, \quad \mathbf{E}^{1}_{2g} = \frac{1}{6}\begin{vmatrix} 0 & \mathbf{d} & 0 \\ -\mathbf{d} & -\mathbf{d} & \sqrt{2}\mathbf{d} \\ 0 & \sqrt{2}\mathbf{d} & 0 \end{vmatrix}, \quad \mathbf{E}^{2}_{2g} = \frac{1}{18}\begin{vmatrix} -\mathbf{d} & 0 & \sqrt{2}\mathbf{d} \\ 0 & 5\mathbf{d} & 0 \\ \sqrt{2}\mathbf{d} & 0 & -2\mathbf{d} \end{vmatrix} \tag{S7}$$

The 480 cm^{-1} Raman band has the A_{1g} symmetry and corresponds to transverse acoustic phonon branch reflecting the singularities in the PDOS of boron nanosheets. This band is Raman active in the $z(x'x')\bar{z}$ backscattering geometry along z||[001], while the incident and scattered light are polarized along x'||[110], and is not Raman active in the $z(x'y')\bar{z}$ backscattering geometry (Fig 4A, main paper), according to Raman tensor (S7) for the A_{1g} mode of the D_{6h} point group. This band is always Raman active in backscattering along z||[111] (the A_{1g} tensor (S4)). The 1230 cm^{-1} Raman band has the E_{1g} symmetry and corresponds to transverse optical phonon branch reflecting the singularities in the PDOS of boron nanosheets. This band is always Raman active in backscattering both along z||[001] and z||[111], according to Raman tensors (S4, 7) for the E_{1g} mode of the D_{6h} point group. Thus, the polarization properties of Raman spectra of boron nanosheets are in agreement with selection rules for the D_{6h} point group.

3. Electronic Raman scattering in BDD

Band num.	Freq. (cm^{-1})	Energy (meV)	Transition
	16	1.98	Δ'
	32	3.97	Δ''
	~47	5.86	Δ'''
	64	7.93	Δ''''
1	92	11.41	$1s(p_{1/2}) \rightarrow 2s(\mathrm{p}_{3/2})$
2	109	13.51	$1s(p_{3/2}) \rightarrow 2s(p_{3/2})$
3	122	15.13	$1s(p_{1/2}) \rightarrow 2s(p_{1/2})$
4	140	17.36	$1s(p_{3/2}) \rightarrow 2s(p_{1/2})$
5	182	22.57	$1s(p_{1/2}) \rightarrow 3s(p_{3/2})$
6	205	25.42	$1s(p_{3/2}) \rightarrow 3s(p_{3/2})$
7	224	27.81	$1s(p_{1/2}) \rightarrow 3s(p_{1/2})$
8	243	30.15	$1s(p_{3/2}) \rightarrow 3s(p_{1/2})$
9	281	34.85	$1s(p_{1/2}) \rightarrow 4s(p_{3/2})$
**10?**	**297**	**36.83**	$\mathbf{1s(p_{3/2}) \rightarrow 4s(p_{3/2})}$
11	343	42.53	$1s(p_{1/2}) \rightarrow 4s(p_{1/2})$
**12?**	**360**	**44.64**	$\mathbf{1s(p_{3/2}) \rightarrow 4s(p_{1/2})}$

Table S3 Frequencies and energies of the Δ'- Δ'''' spin-orbit splitting of the ns boron acceptor states and Lyman electronic transitions from the $1s(p_{3/2})$ and $1s(p_{1/2})$ ground states to the n$s(p_{3/2})$ and n$s(p_{1/2})$ excited states in the boron doped diamond.

4. Simple calculation of boron content from the structure model

Calculation of the boron concentration was done in the assumption that the modulation period Λ is equal to 43.26 Å (2.06 Å × 21) in all cases within consideration, where 2.06 Å is the interplanar distance for diamond in [111] direction. The length of the main diamond diagonal is 2.06 × 3 = 6.18 Å where 3 relates to the ABC layers in the diamond unit cell.

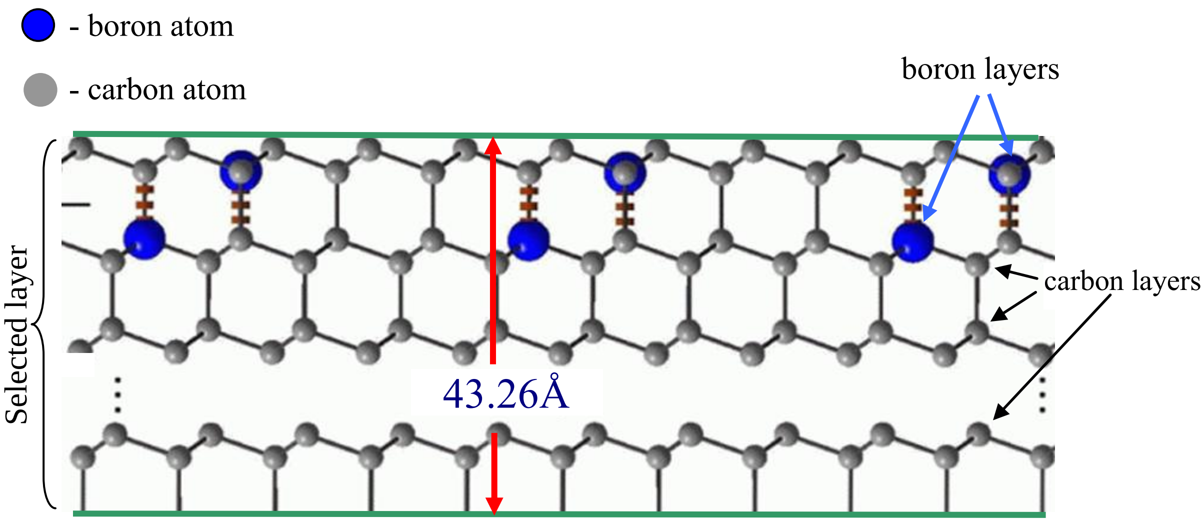

Fig. S5

The total boron concentration (at%) can be estimated as the concentration of the boron in the selected layer in the periodic structure shown in Fig. S5. Below is the calculation of boron concentration depending on the distance between boron atoms in the hexagon (see Fig. 3B, main paper). Displacement of boron atoms in the calculations are not taken into account. The calculation for selected layer is performed with the formula

$$K_V = m \times K_B / n, \quad \text{(S8)}$$

where K_V is the total concentration (at%), K_B is the boron concentration within the layer, m is the number of layers with the boron and carbon atoms, n is the total number of layers.

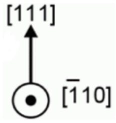

Hexagon with boron atoms

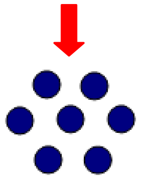

1) All atoms in the layer are borons

$K_B = 1$, $m = 2$, $n = 42$

$K_V = 2\times1/42 = 1/21 = 0.0476$ or 4.76 at%.

This case cannot be realized. It is impossible to grow a single crystal.

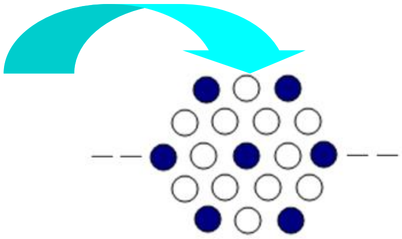

2) One carbon atom locates between the boron atoms in the hexagon.

$K_B = 3/12 = 1/4$, $m = 2$, $n = 42$

$K_V = 2\times1/4/42 = 0.0119$ or ~1.2 at %.

Three is the number of boron atoms in the upper and bottom layer. It should be noted that for the selected hexagonal cell there are six corner atoms and one atom inside the hexagonal unit cell. Between the upper layer and the bottom layer with the boron and carbon atoms there are 40 layers with carbon atoms only. The whole number of layers n = 42.

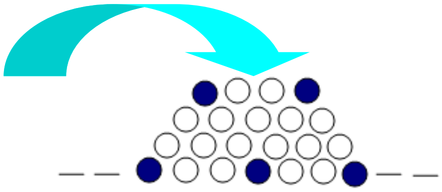

3) Two carbon atoms locate between the boron atoms in the hexagon.

$K_V = 3/27 \times 2/42 = 0.0053$ or 0.53 at %.

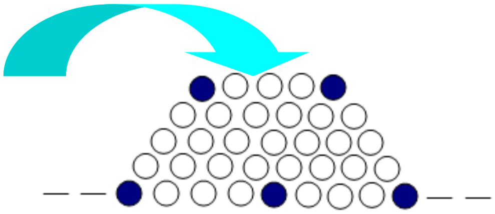

4) Three carbon atoms locate between the boron atoms in the hexagon.

$K_V = 3/48 \times 2/42 = 0.00298$ or ~0.3at%.

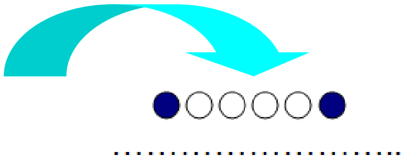

5) Four carbon atoms locate between the boron atoms in the hexagon.

$K_V = 3/75 \times 2/42 = 0.00227$ or ~0.23 at%.

6) Five carbon atoms locate between the boron atoms in the hexagon.

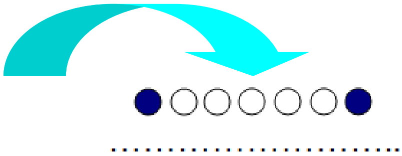

$K_V = 3/108 \times 2/42 = 0.0013$ or 0.13 at%.

7) Six carbon atoms locate between the boron atoms in the hexagon.

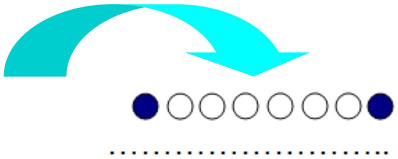

$K_V = 3/147 \times 2/42 = 0.00097$ or 0.097 at%.

8) Similar calculations can be done for the rest cases.

Dependence of the boron concentration on the modulation period can be estimated with the formula (S8). In the extreme case the modulation period is equal to 6.18 Å, i.e. the length of main diagonal of diamond unit cell with AA′BB′CC′ sequence of layers, where n = 6 (1.54 Å+0.52 Å +1.54 Å+0.52 Å +1.54 Å+0.52 Å = 6.18 Å) or simple ABC (2.06 Å×3 = 6.18 Å). For the case 2), when n=6 (Λ=6.18 Å), the boron concentration is $K_V=2\times1/4/6\approx 8.4\%$.

References

1. Warren JL, Yarnell JL, Dolling G, and RA Cowley. Lattice Dynamics of Diamond. Phys. Rev. 1967;158:805.
2. Tubino R, Piseri L, and Zerbi G, Lattice dynamics and spectroscopic properties by a valence force potential of diamond-like crystals: C, Si, Ge, Sn. J. Chem. Phys. 1972;56:1022.
3. Pavone P, Karch K, Schütt O, Strauch D, Windi W, Giannozzi P, and Baroni S. *Ab initio* lattice dynamics of diamond. Phys. Rev. B 48, (1993), 3156.
4. Cardona M. Resonance Phenomena In: Cardona M and Güntherodt G, editors. Light Scattering in Solids II. Springer-Verlag Berlin Heidelberg New York, 1982. p. 19-173.
5. Blank VD, Denisov VN, Kirichenko AN, Kuznetsov MS, Mavrin BN, Nosukhin SA, Terentiev SA. Raman scattering by defect induced excitations in boron-doped diamond single crystals. Diamond Relat. Mater. 2008;17:1840.
